# A review of *Solenysa* spiders from Japan (Araneae, Linyphiidae), with a comment on the type species *S. mellotteei* Simon, 1894

**DOI:** 10.3897/zookeys.481.8545

**Published:** 2015-02-04

**Authors:** Fang Wang, Hirotsugu Ono, Lihong Tu

**Affiliations:** 1 College of Life Sciences, Capital Normal University, 105, Xisanhuanbeilu Str., Haidian Dist., Beijing, 100048, P. R. China; 2 Department of Zoology, National Museum of Nature and Science, 4-1-1, Amakubo, Tsukuba-shi, Ibaraki, 305-0005, Japan

**Keywords:** Genitalia, morphology, new species, taxonomy

## Abstract

The present paper gives a review of *Solenysa* species from Japan and provides a solution for the species bearing the generotype name *Solenysa mellotteei* Simon, 1894. A total of six species are recorded, including two new species *Solenysa macrodonta* **sp. n.** and *Solenysa trunciformis* **sp. n.** The species collected from Kawasaki (NSMT-Ar 11154) and Hachioji should be the generotype *Solenysa mellotteei*, with *Solenysa akihisai* Tu, 2011, **syn. n.** as its junior synonym. To distinguish these congeneric species from each other, their genital characters are provided in detail based on images collected by scanning electron microscopy and light microscopy.

## Introduction

The spider genus *Solenysa* was erected by Simon (1894) to accommodate the linyphiid species, *Solenysa mellotteei* Simon, 1894, which was collected from Japan by a French diplomat, A. Mellottée. Other *Solenysa* species were described successively from other places in Japan, the Chinese mainland, Taiwan, and the Korean Peninsula (see review by Tu and Li 2006). In recent studies, several new species were sorted from the *Solenysa* collections deposited in the Department of Zoology, National Museum of Nature and Science (ex National Science Museum, Tokyo), Japan (Tu et al. 2007, Tu and Hormiga 2011, Ono 2011). Prior to this study, there were five *Solenysa* species reported from Japan: *Solenysa mellotteei* Simon, 1894 (type locality: Yokohama, Kanagawa Prefecture), *Solenysa akihisai* Tu, 2011 (type locality: Hachioji, Tokyo), *Solenysa ogatai* Ono, 2011 (type locality: Okazaki-shi, Aichi Prefecture), *Solenysa partibilis* Tu, Ono & Li, 2007 (type locality: Mt. Ibuki-yama, Shiga Prefecture) and *Solenysa reflexilis* Tu, Ono & Li, 2007 (type locality: Itsuki-mura, Kumamoto Prefecture). According to results of a phylogenetic analysis based on morphological data, the twelve known *Solenysa* species were divided into four groups, and the four species from Japan share a complex of genital characters, forming the *Solenysa mellotteei* group (Tu and Hormiga 2011).

As more species were recognized, a problem regarding the type species of *Solenysa* emerged. Generally, the *Solenysa* species occurring in Japan are endemic, have a restricted distribution with little overlap (Fig. 7). Small in body size, similar in somatic features and genital morphology, it is difficult to distinguish them from each other without examining their genitalia in detail (Tu and Hormiga 2011). Consequently, all *Solenysa* spiders collected from the islands of Japan have long been identified as *Solenysa mellotteei* (Oi 1960, Yaginuma 1986, Irie and Saito 1987, Chikuni 1989, Lee et al. 2004, Tu and Li 2006). Redescriptions for the species currently bearing the generotype name, *Solenysa mellotteei*, in reviews of *Solenysa* were not based on the type material (Tu and Li 2006, Tu and Hormiga 2011) and the species are different from those collected from the places more adjacent to the inferred type locality (Ono 2011). It is necessary to make a review to distinguish the species of the *Solenysa mellotteei* group and to establish the identity of the generotype *Solenysa mellotteei*. From the materials collected throughout the islands of Japan, we identified six species in total, including two new species and one new synonymy. In the present study, all these *Solenysa* spiders were studied by using scanning electric microscopy (SEM) and light microscopy to show genital characters in detail. Descriptions for the new species and redescriptions for the known species are presented.

## Materials and methods

Specimens were examined and measured by using a Leica MZ16A stereo microscope. Further details, such as epigynes, were studied with a Leica DM5500B compound microscope. Digital images were taken with a Leica DFC 500 camera and as a composite of multiple focus images assembled using the software package Leica Application Suite. Epigynes were cleared in methyl salicylate (Holm 1979) for examination under the microscope and temporarily mounted as described by Grandjean (1949) and Coddington (1983). SEM images were taken by using a Hitachi S-3400N scanning electron microscope at China Agriculture University. For SEM examination, the specimens were prepared as described by Álvarez-Padilla and Hormiga (2008). The non-chitinous abdominal tissue was digested with Sigma Pancreatin LP 1750 enzyme complex to expose the internal structures for examination. Due to the unavailability of specimen, no SEM image provided for the male palp of *Solenysa reflexlis*.

All measurements are given in millimeters. The leg measurements are given in the following sequence: Total (femur, patella+tibia, metatarsus, tarsus). Terminology for the genital characters follows Tu and Hormiga (2011). The specimens examined here have been deposited in the Department of Zoology, National Science Museum, Tokyo, Japan (NSMT) and in College of Life Sciences, Capital Normal University, Beijing (China).

### Anatomical abbreviations used in the text and figures

**Male palp**

ATA anterior terminal apophysis

DSA distal suprategular apophysis

E embolus

LC lamella characteristica

LC1 anterior branch of LC

LC2 median branch of LC

LC3 posterior branch of LC

MTA median terminal apophysis

P paracymbium

PBP cymbial probasal process

PTA posterior terminal apophysis

R radix

STT
*solenysa* tegular triangle

T tegulum

**Epigyne**

CG copulatory groove

CO copulatory opening

DP dorsal plate

EC epigynal collar

FG fertilization groove

S spermatheca

SL solenoid

VP ventral plate

## Taxonomy

### Linyphiidae Blackwall, 1859

#### Solenysa

Taxon classificationAnimaliaAraneaeLinyphiidae

Simon, 1894

##### Type species.

*Solenysa mellotteei* Simon, 1894.

##### Composition.

Fourteen species including two new species: *Solenysa geumoensis* Seo, 1996, *Solenysa lanyuensis* Tu, 2011, *Solenysa longqiensis* Li & Song, 1992, *Solenysa macrodonta* sp. n., *Solenysa mellotteei* Simon, 1894, *Solenysa ogatai* Ono, 2011, *Solenysa partibilis* Tu, Ono & Li, 2007, *Solenysa protrudens* Gao, Zhu & Sha, 1993, *Solenysa reflexilis* Tu, Ono & Li, 2007, *Solenysa retractilis* Tu, 2011, *Solenysa tianmushana* Tu, 2011, *Solenysa trunciformis* sp. n., *Solenysa wulingensis* Li & Song, 1992 and *Solenysa yangmingshana* Tu, 2011.

##### Diagnosis.

**Figure 1.**
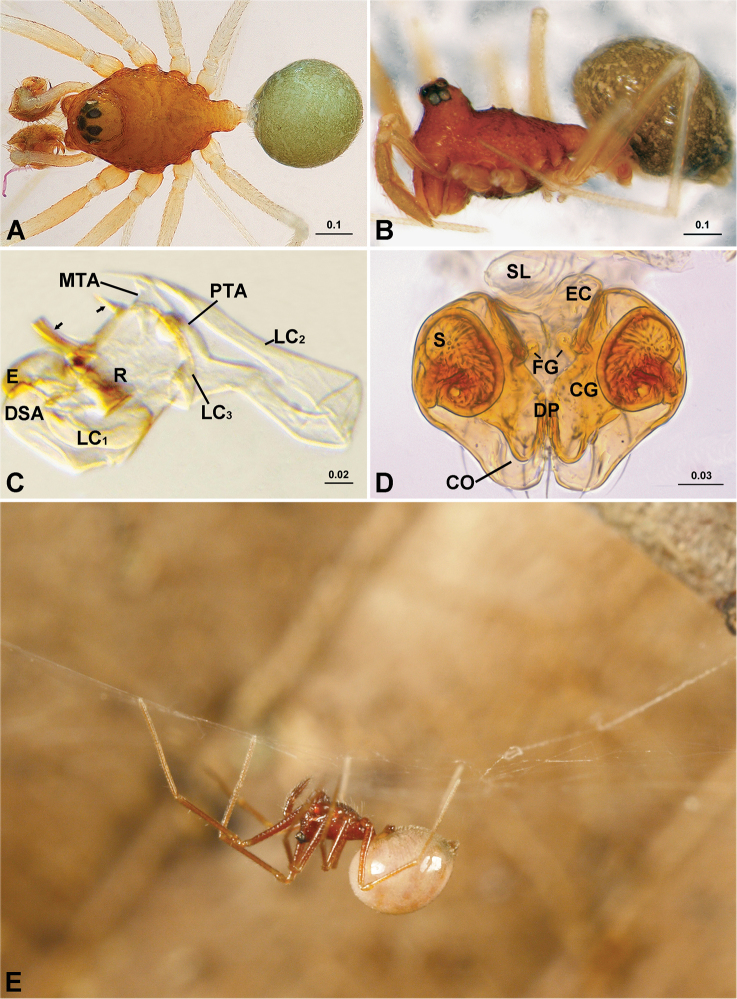
*Solenysa trunciformis* sp. n. (**A–D**) and *Solenysa partibilis* (**E**). **A** male, dorsal **B** female, lateral **C** male palpal embolic division, ventral, arrows indicate two anterior protrusions of MTA **D** epigyne, dorsal **E** female, lateral in living state, showing non-functional state of epigyne. **CO** copulatory opening; **CG** copulatory groove; **DP** dorsal plate; **DSA** distal suprategular apophysis; **E** embolus; **EC** epigynal collar; **FG** fertilization groove; **LC** lamella characteristica; **LC**_1_ anterior LC branch; **LC_2_** median LC branch; **LC_3_** posterior LC branch; **MTA** median terminal apophysis; **PTA** posterior terminal apophysis; **R** radix; **S** spermatheca; **SL** solenoid. Photo of *Solenysa partibilis* provided by Akihisa Andoh. [Scale bars: mm]

**Figure 2.**
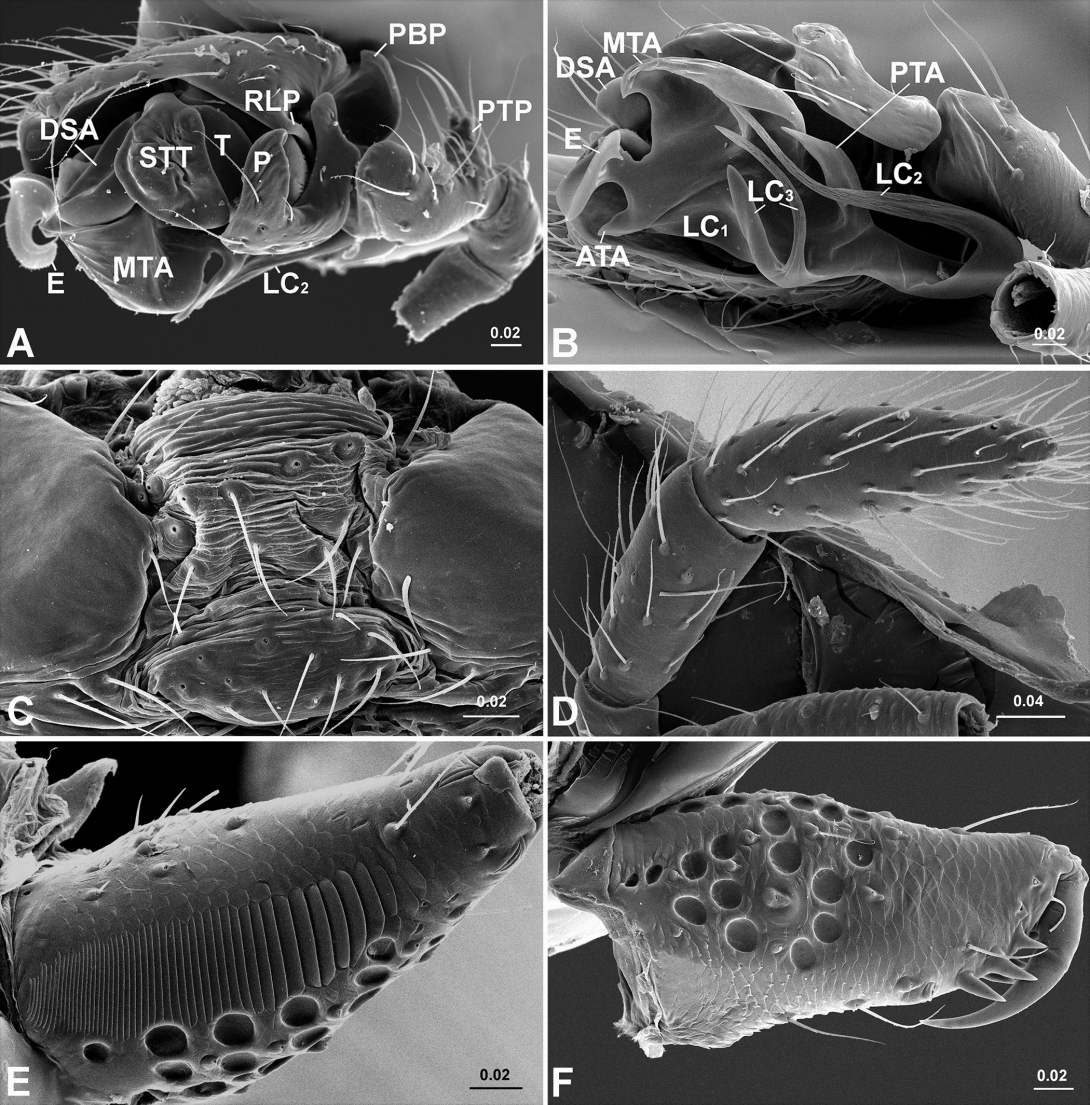
*Solenysa mellotteei*. **A** male palp, retrolateral **B** ditto, ventral **C** anterior part of male abdomen, ventral, shows epiandrous fusules absent and smooth book lung cover **D** female palp, shows tarsus claw absent **E** male chelicera, ectal, shows stridulatory striae **F** female chelicerae. **ATA** anterior terminal apophysis; **DSA** distal suprategular apophysis; **E** embolus; **LC** lamella characteristica; **LC_1_** anterior LC branch; **LC_2_** median LC branch; **LC_3_** posterior LC branch; **MTA** median terminal apophysis; **P** paracymbium; **PBP** probasal cymbial process; **PTA** posterior terminal apophysis; **PTP** proximal tibial process; **RLP** cymbial retrolateral process; **STT** *Solenysa* tegular triangle; **T** tegulum. [Scale bars: mm]

*Solenysa* species can be distinguished from all other linyphiids by the four lobes at the sides of carapace, the rounded pits scattered on the carapace and the tubular-shaped petiole (Fig. 1A–B). Females are also diagnosed by the presence of a long membranous solenoid, connecting between the epigyne and the abdomen (Fig. 1D), males by the presence of *Solenysa* tegular triangle in male palp (Fig. 2A).

##### Description.

See Tu and Li (2006) and Tu and Hormiga (2011).

##### Distribution.

Japan, Chinese mainland, Taiwan, Korea.

##### Comments.

The subfamily placement of *Solenysa* remains controversial as its complex type of male palp with well developed lamella characteristica and terminal apophysis is like those in Micronetinae Hull, 1920, but the simple type of epigyne is like those in Erigoninae Emerton, 1882. Based on the movable epigyne, Saaristo (2007) included it in his new subfamily Ipainae Saaristo, 2007. However, the results of a phylogenetic analysis of Linyphiidae queried the monophyly of “ipaines”, and suggested that “micronetines” and erigonines form a monophyletic group (Arnedo et al. 2009). Furthermore, the results of a phylogenetic analysis of erigonines based on morphological data showed that all *Solenysa* species form a monophyly robustly supported by a long list of synapomorphies, and other synapomorphies suggested its close relationship with erigonines although its sister group remained unresolved (Tu and Hormiga 2011). Accordingly, the well-developed lamella characteristica and terminal apophysis in *Solenysa* should be regarded as homologous to those of “micronetines” and secondarily lost in erigonines; their simple type epigyne also derived from the complex type of “micronetines”. The morphology of solenoid in *Solenysa* is different from the extensive basal parts in *Acanoides beijingensis* Sun, Marusik & Tu, 2014 and *Acanoides hengshanensis* (Chen & Yin, 2000) (Sun et al. 2014: figs 4G, 5G), and in *Wubanoides uralensis* (Pakhorukov, 1981), *Epibellowia enormita* (Tanasevitch, 1988) and *Epibellowia septentrionalis* (Oi, 1960) (Tanasevitch 1996: figs 7–9). Whether the movable epigyne has a single origin or independently evolved multiple times in linyphiids needs to be tested in future studies.

A phylogenetic analysis based on morphological data (Tu and Hormiga 2011) suggested that the twelve known *Solenysa* species are divided into four clades. Among them, the four species occurring in Japan formed a monophyletic clade, unambiguously supported by the following synapomorphies: the presences of hook shaped cymbial probasal process, half rounded *Solenysa* tegular triangle and copulatory grooves enter the spermathecae from the outer sides.

#### Solenysa mellotteei group

Taxon classificationAnimaliaAraneaeLinyphiidae

Tu & Hormiga, 2011

##### Composition.

Six species: *Solenysa mellotteei* Simon, 1894, *Solenysa macrodonta* sp. n., *Solenysa ogatai* Ono, 2011, *Solenysa partibilis* Tu, Ono & Li, 2007, *Solenysa reflexilis* Tu, Ono & Li, 2007 and *Solenysa trunciformis* sp. n.

##### Diagnosis.

**Figure 3.**
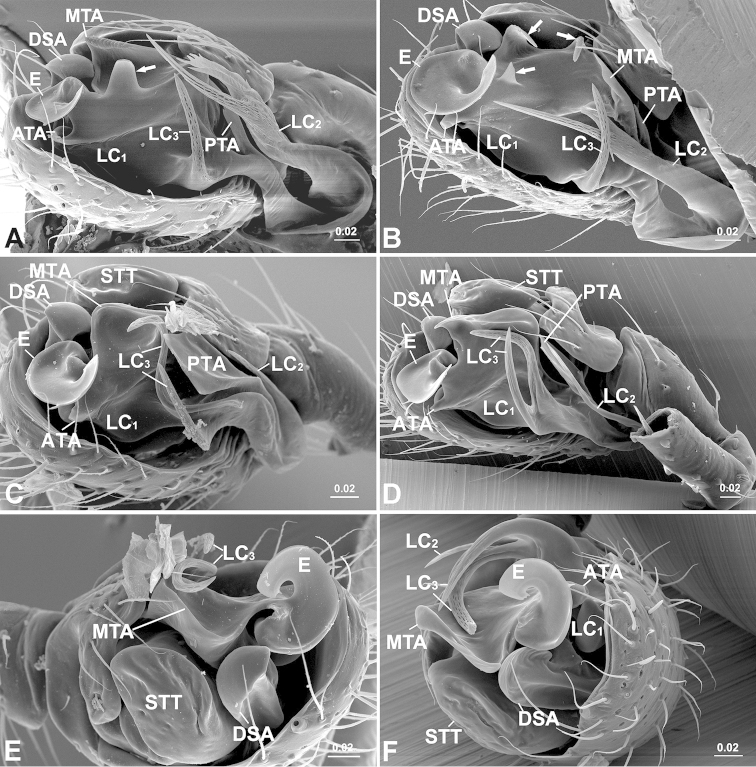
Male palpal embolic division. **A** *Solenysa macrodonta* sp. n., ventral, arrow indicates central tooth **B** *Solenysa trunciformis* sp. n., ventral, arrows indicate central tooth and two anterior protrusions of MTA **C** *Solenysa ogatai*, ventral **D** *Solenysa partibilis*, ventral **E** *Solenysa ogatai*, anterior **F** *Solenysa partibilis*, anterior. **ATA** anterior terminal apophysis; **DSA** distal suprategular apophysis; **E** embolus; **LC** lamella characteristica; **LC_1_** anterior LC branch; **LC_2_** median LC branch; **LC_3_** posterior LC branch; **MTA** median terminal apophysis; **PTA** posterior terminal apophysis; **STT** *Solenysa* tegular triangle. [Scale bars: mm]

**Figure 4.**
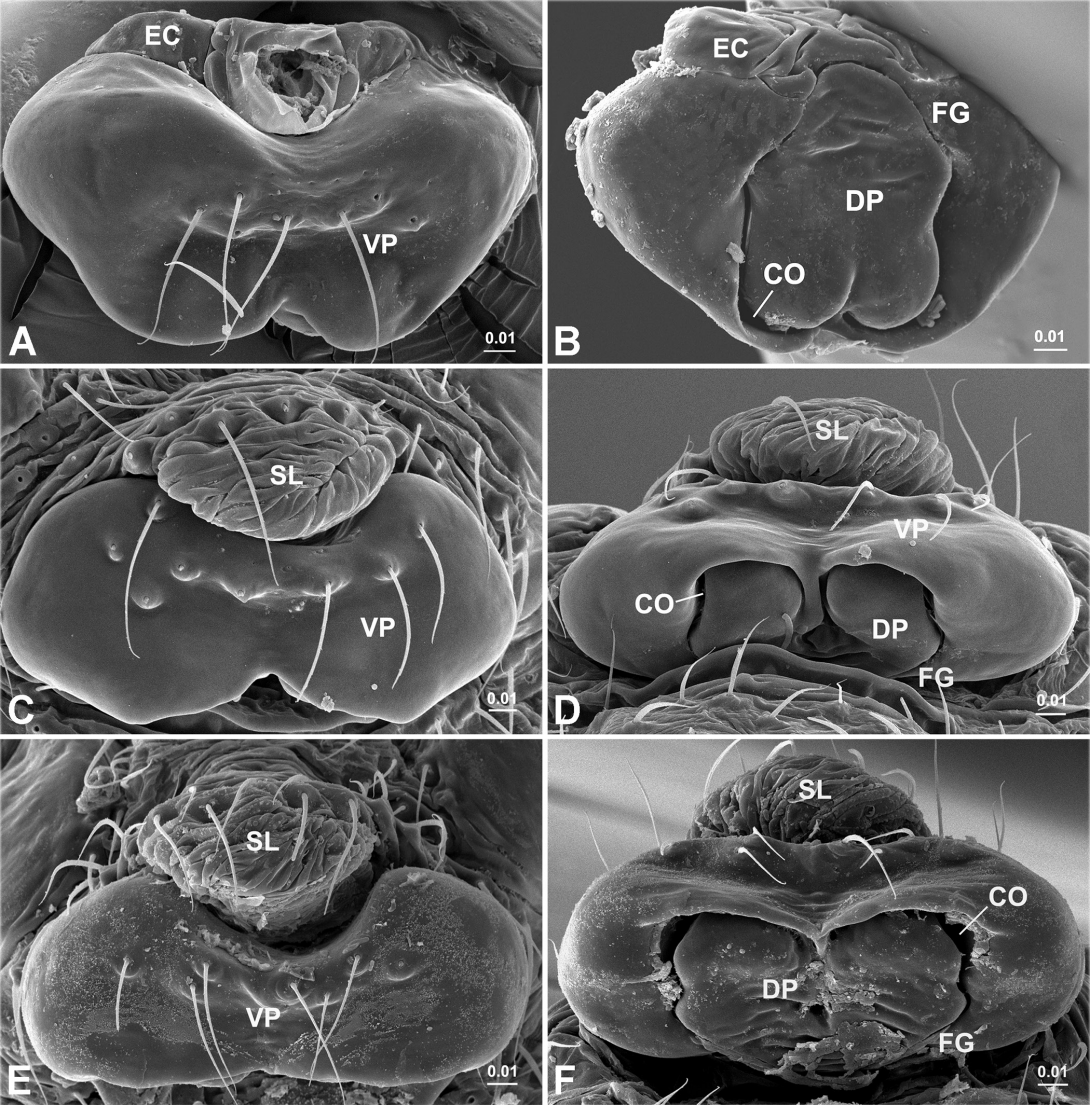
Epigyne. *Solenysa mellotteei* (**A**–**B**), *Solenysa macrodonta* sp. n. (**C**–**D**), *Solenysa reflexilis* (**E–F**). **A, C, E** ventral, **A** with epigynal collar removed **B, D, F** dorsal. **CO** copulatory opening; **DP** dorsal plate; **EC** epigynal collar; **FG** fertilization groove; **VP** ventral plate; **SL** solenoid. [Scale bars: mm]

**Figure 5.**
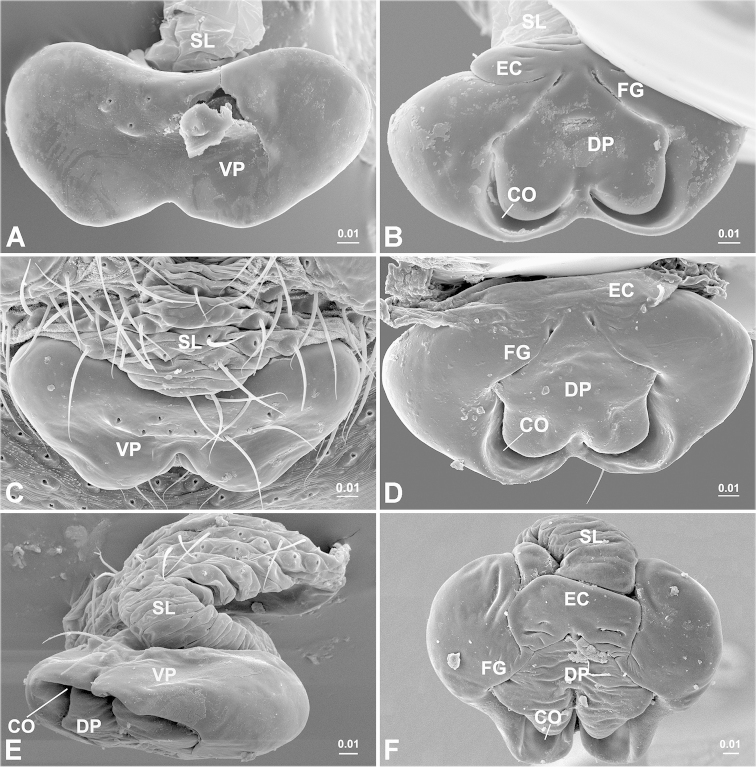
Epigyne. *Solenysa ogatai* (**A–B**), *Solenysa partibilis* (**C–D**), *Solenysa trunciformis* sp. n. (**E–F**). **A, C** ventral **B, D, F**, dorsal **E** lateral, with solenoid artificially loosened. **CO** copulatory opening; **DP** dorsal plate; **EC** epigynal collar; **FG** fertilization groove; **VP** ventral plate; **SL** solenoid. [Scale bars: mm]

Males of *Solenysa mellotteei* group are distinguished from all other three groups by the spiral plate-shaped embolus (Fig. 3E), the hook-shaped cymbial probasal process and by the half rounded *Solenysa* tegular triangle (Fig. 2A). Females are characterized by the dorsoventrally folded solenoid (Figs 4C, 5C), the spherical spermathecae and the pocket shaped copulatory grooves entering the spermathecae from the outer sides (Fig. 1D).

##### Description.

All *Solenysa* species have quite uniform somatic morphology. Somatic characters as in the genus description (see also Tu and Li 2006, Tu and Hormiga 2011).

**Figure 6.**
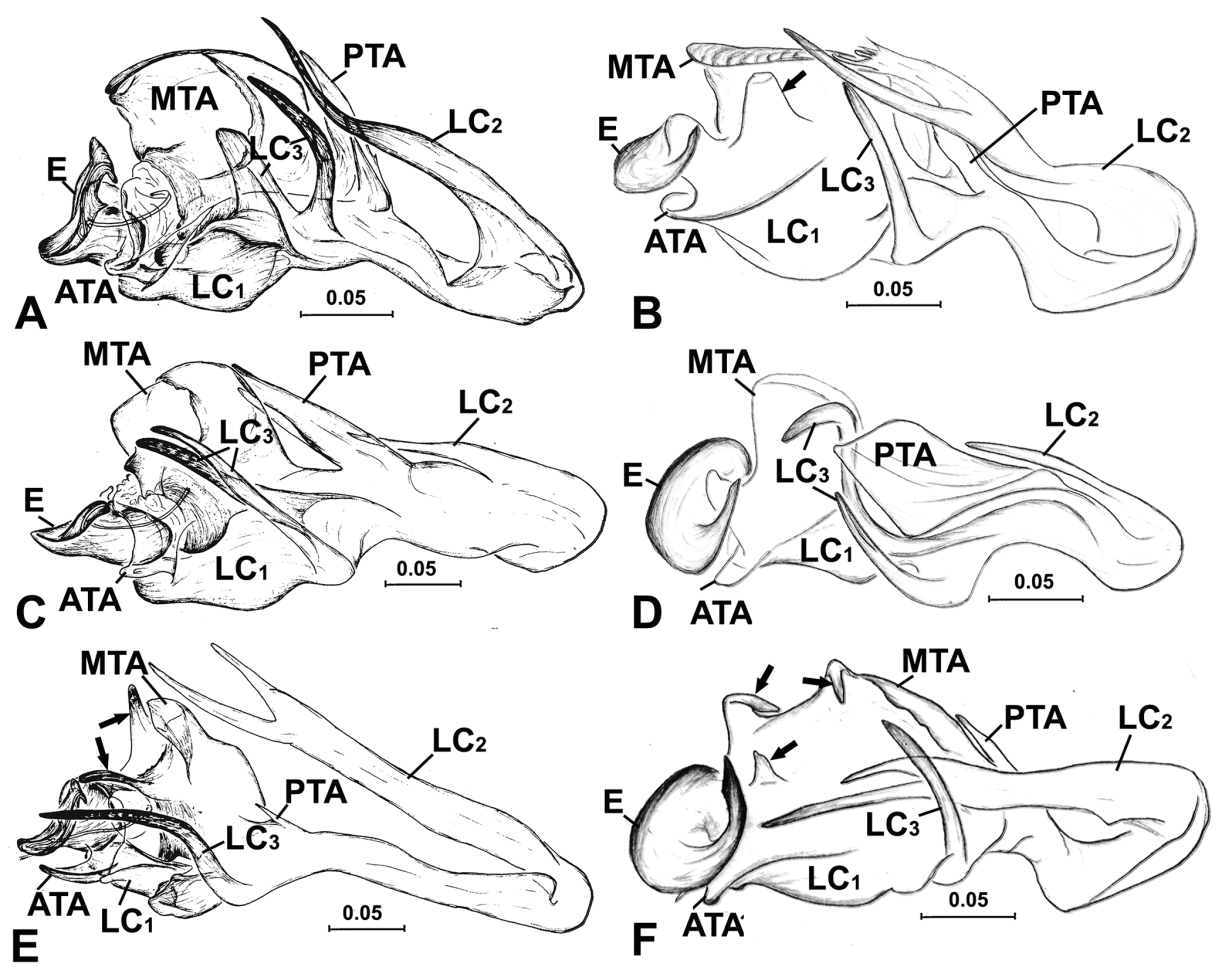
Male palpal embolic division, ventral. **A** *Solenysa mellotteei* **B** *Solenysa macrodonta* sp. n., arrows indicate central tooth **C** *Solenysa partibilis* **D** *Solenysa ogatai* **E** *Solenysa reflexilis*, arrows indicate two anterior protrusions **F** *Solenysa trunciformis* sp. n., arrows indicate central tooth and two anterior protrusions of MTA. **ATA** anterior terminal apophysis; **E** embolus; **LC** lamella characteristica; **LC_1_** anterior LC branch; **LC_2_** median LC branch; **LC_3_** posterior LC branch; **MTA** median terminal apophysis; **PTA** posterior terminal apophysis; **STT** *Solenysa* tegular triangle. [Scale bars: mm]

Male palp (Fig. 2A–B). Tibia twice as long as patella, with proximal process furnished by two long bristles. Cymbium with hook-like proximal process and small retrolateral process, forming articulation with proximal arm of U-shaped paracymbium. Tegulum with half rounded *Solenysa* tegular triangle and stout distal suprategular apophysis. Embolic division (Fig. 6): embolus spiral plate shaped with two apophyses, one at outer margin, and one distally (Fig. 3E). Radix embedded within membranous area connecting terminal apophysis and lamella characteristica (Figs 1C, 2B). Terminal apophysis divided into three parts, with median one as enlarged sclerite. Lamella characteristica with three well-developed branches, anterior branch (LC_1_) stout and extending forward, following embolus trajectory; median one (LC_2_) long and slender, dragging backwards and pointing forward, bifid in some species (Fig. 3A); posterior one (LC_3_) sharp and strongly sclerotized, bifid in some species (Fig. 3B).

Epigyne (Figs 1D, 4A–B). Strongly sclerotized box-shaped, having a well-developed epigynal collar at anterior part connecting with solenoid. Solenoid flexible, dorsoventrally folded (Fig. 5C) in non-functional stage, holding epigyne up (Fig. 1E). Spermathecae large spherical. Copulatory openings as a pair of crescent shaped slits hidden on dorsal surface. Copulatory grooves enlarged into half round pocket-shaped, matching to spiral plate-shaped embolus, entering spermathecae outboard. Fertilization grooves convergent, extending forward.

##### Distribution.

**Figure 7.**
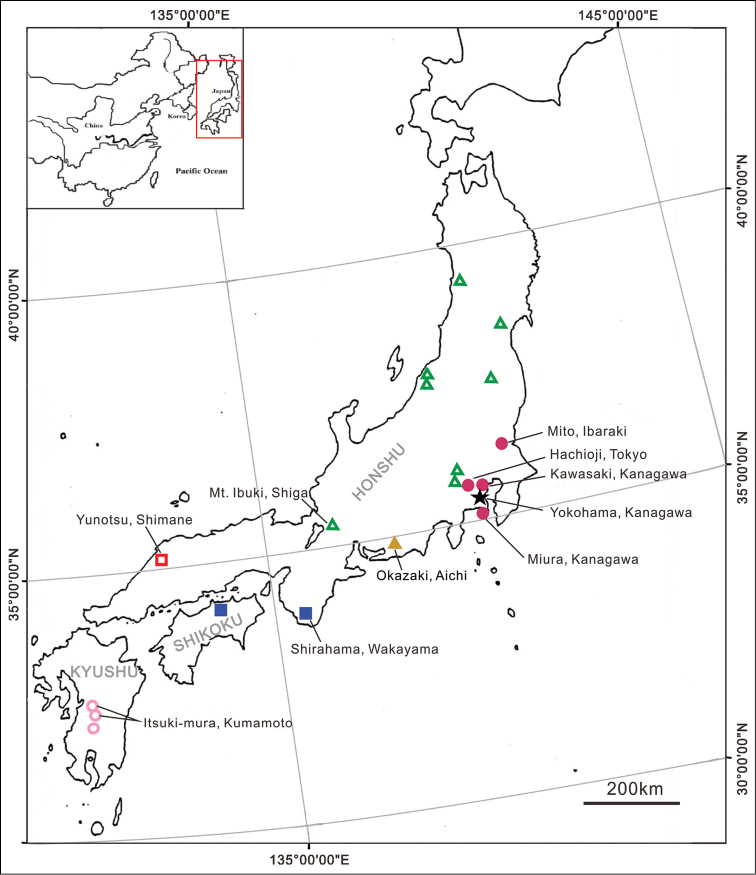
Collecting locations of *Solenysa* species from Japan. ● *Solenysa mellotteei* □ *Solenysa macrodonta* sp. n. ▲ *Solenysa ogatai* △ *Solenysa partibilis* ○ *Solenysa reflexilis* ■ *Solenysa trunciformis* sp. n. ★ type locality of *Solenysa mellotteei*.

Japan (Honshu, Shikoku, Kyushu, Fig. 7).

#### Solenysa mellotteei

Taxon classificationAnimaliaAraneaeLinyphiidae

Simon, 1894

Figs 2 , 4A–B , 6A

Solenysa mellottei Simon, 1894: 677; Lee et al. 2004: 100; Ono et al. 2009: 330, figs 1087–1091.
Solenysa mellotteei : Yaginuma 1986: 78, fig. 42.2; Irie and Saito 1987: 23, fig. 21; Chikuni 1989: 56, fig. 48.
Solenysa akihisai : Tu and Hormiga 2011: 499, fig. 8A–I.

##### Material examined.

1♂ and 1♀ (NSMT-Ar 11154), Japan, Honshu, Kanagawa Prefecture, Kawasaki-shi, Asao-ku, Kurokawa, 35°32'N, 139°43'E, 15 Nov. 1997, coll. Mitsuru Ban; 1♂ and 2♀♀, Japan, Honshu, Tokyo, Hachioji, 35°42'N, 139°18'E, 20 Dec. 2003, coll. Akihisa Andoh; 3♂♂ and 3♀♀ (CNU-J02), Japan, Honshu, Ibaraki Prefecture, Mito-shi, Tara, 36°24.35'N, 140°24.55'E, 27 Nov. 2000, coll. Akihisa Andoh; 3♂♂ and 7♀♀, Japan, Honshu, Tokyo, Hachioji, Kamikawa, 35°42.55'N, 139°15.23'E, alt. 230 m, 9 Nov. 2008, coll. Akihisa Andoh; 5♂♂ and 2♀♀ (CNU-J22), Japan, Honshu, Kanagawa Prefecture, Miura, Ko-ajiro, 35°09.88'N, 139°37.65'E, alt. 20 m, 1 Mar. 2008, coll. Akihisa Andoh; 2♂♂ (CNU-J32), Japan, Honshu, Ibaraki Prefecture, Mito, Tano, 36°24.55'N, 140°24.38'E, alt. 45 m, 13 Jun. 2009, coll. Akihisa Andoh.

##### Diagnosis.

*Solenysa mellotteei* is similar to *Solenysa partibilis* and *Solenysa ogatai* in male palps having the posterior branch of lamella characteristica (LC_3_) divided into two parts (Fig. 6A, C, D), and in females having an apple-shaped epigyne. Males can be distinguished by: the anterior part of LC_3_ is flag-shaped in *Solenysa mellotteei* (Fig. 2B), long spike-shaped in *Solenysa ogatai* (Fig. 3C) and *Solenysa partibilis* (Fig. 3D); the posterior part of LC_3_ S-curved in *Solenysa ogatai* (Fig. 3C), L-curved in *Solenysa partibilis* (Fig. 3D). Females can be distinguished by the inverse triangular epigynal collar and the dorsal plate as wide as long in *Solenysa mellotteei* (Fig. 4A, Tu and Hormiga 2011: fig. 8I), the dorsal plate wider than long in *Solenysa partibilis* and *Solenysa ogatai* (Fig. 5B, D), and the epigynal collar more than four times wider than long in *Solenysa ogatai* (Fig. 5B), less than twice wider than long in *Solenysa partibilis* (Tu and Hormiga 2011: fig. 11I).

##### Description.

*Solenysa mellotteei* has somatic morphology typical of *Solenysa* (Fig. 1A, B, E) and a genital pattern of the *Solenysa mellotteei* group (Fig. 2A–B). For somatic and genital characters, see the description provided by Tu and Hormiga (2011) for *Solenysa akihisai*, the junior synonym of *Solenysa mellotteei*.

##### Distribution.

Japan (Honshu, Fig. 7).

##### Comments.

The problem with the identification of the generotype *Solenysa mellotteei* arose because *Solenysa* species occurring in Japan, previously all identified as *Solenysa mellotteei*, are now distinguished as six species. Since most of them have restricted distributions without any overlap (Fig. 7), it has long remained ambiguous which species is the original *Solenysa mellotteei* described by Simon (1894). The type material of *Solenysa mellotteei* was not located (Tu and Li 2006), and the original description by Simon (1894) did not provide detailed information about the type locality. According to Ono (2011), the French diplomat A. Mellottée, who had spent only two years in Japan, stayed in the foreign settlement at Yokohama and collected spiders in the surrounding area. All his collections were contributed to the National Museum of Natural History, Paris (Ono 1987, Takahashi 2000) and studied by Simon (1886a, 1886b, 1889, 1893, 1894, 1895). For that reason, Ono (2011) inferred the type locality of *Solenysa mellotteei* should be Yokohama, Kanagawa Prefecture. In the first review of the genus by Tu and Li (2006), the redescription of *Solenysa mellotteei* was based on a pair of specimens sent by a Japanese scholar and did not include any collecting data. In the phylogenetic revision of *Solenysa* (Tu and Hormiga 2011), the supplementary material of the same species did not come from the type locality, but from Esuzaki, Susami-cho, Wakayama Prefecture. However, specimens collected from Hachioji, Tokyo, which is much closer to the type locality (Fig. 7), were proposed as a new species *Solenysa akihisai*. In the present study we examined material collected from three localities adjacent to Yokohama: Hachioji, Kawasaki (NSMT-Ar 11154) and Miura (Fig. 7), as well as specimens from Mito, and found that they are the same species, which should bear the generotype name *Solenysa mellotteei*, and *Solenysa akihisai* is a junior synonym of it. The materials collected from Wakayama Prefecture, and those from Shikoku Island are proposed here as a new species *Solenysa trunciformis* sp. n.

#### Solenysa macrodonta sp. n.

Taxon classificationAnimaliaAraneaeLinyphiidae

http://zoobank.org/E937495C-A852-4FB6-8739-F40F5AA5C1E8

Figs 3A , 4C–D

##### Types.

Male holotype (CNU-J21), Japan, Honshu, Shimane Prefecture, Yunotsu, Nishida, 35°05.06'N, 132°24.10'E, 27 Jul. 2006, coll. Akihisa Andoh. Paratype, 1♀, same data as holotype.

##### Diagnosis.

The male palp of *Solenysa macrodonta* sp. n. is similar to those of *Solenysa trunciformis* sp. n. and *Solenysa refrexilis* in the presence of a central tooth at the membranous area embedded the radix (Figs 1C, 3A, 6B), the forked apex of the median branch of lamella characteristica and the long spike-shaped posterior branch (Fig. 3A–B). They can be distinguished from each other by the median part of terminal apophysis, which has a serrate margin in *Solenysa macrodonta* sp. n. (Fig. 3A), but with two anterior protrusions in *Solenysa trunciformis* sp. n. (Fig. 3B) and *Solenysa refrexilis* (Tu et al. 2007: fig. 1D), which is truncate in the former species and pointed in the latter species. The short epigyne of *Solenysa macrodonta* sp. n. is similar to those of *Solenysa partibilis* and *Solenysa reflexilis*, having the dorsal plate wider than long (Fig. 5D). They can be distinguished from each other by the maximum width in ventral view; at the anterior part in *Solenysa partibilis* (Fig. 5C), in the middle in *Solenysa macrodonta* sp. n. (Fig. 4C), and posterior in *Solenysa reflexilis* (Fig. 4E), which also has a straight posterior margin.

##### Description.

Male holotype. Total length 1.33. Carapace, 0.8 long, 0.48 wide. Abdomen, 0.53 long, 0.38 wide. Chelicera with four promarginal and two retromarginal teeth. Length of legs: I 2.53 (0.68 + 0.80 + 0.58 + 0.47); II 2.25 (0.60 + 0.66 + 0.50 + 0.49); III 1.69 (0.47 + 0.50 + 0.39 + 0.33); IV 1.98 (0.61 + 0.64 + 0.43 + 0.30). Tm I: 0.23, Tm IV absent. Measurements for the female were not possible since the single specimen was prepared for SEM examination. Other somatic characters are as in the genus description (Fig. 1A, B, E; see also Tu and Li 2006, Tu and Hormiga 2011).

Male palp (Fig. 3B). General male palpal characters are as in the description for the *Solenysa mellotteei* group. Embolic division (Fig. 6B): radix embedded in the central membranous area connecting with terminal apophysis and lamella characteristica, from where a central tooth protrudes. Median part of terminal apophysis as large sclerite with serrated margin. Anterior branch of lamella characteristica reduced, stout and extending forward following embolus; the median branch ribbon-like, long and slender, dragging backwards, then folding forward, with forked apex, one sharp, one with threaded margin; the posterior long spike-shaped and strongly sclerotized.

Epigyne (Fig. 4C–D). Twice as wide as long in ventral view, with maximum width in the middle. Posterior margin centrally incised. Dorsal plate wider than long.

##### Etymology.

The species name is based on the Latin ‘*macrodontus*’ in reference to the large central tooth protruding from the membranous area connecting with terminal apophysis and lamella characteristica (Fig. 3A).

##### Distribution.

Japan (Honshu, Fig. 7).

#### Solenysa ogatai

Taxon classificationAnimaliaAraneaeLinyphiidae

Ono, 2011

Figs 3C, E , 5A–B

Solenysa ogatai Ono, 2011: 126, figs 11–17.

##### Types.

Male holotype (NSMT-Ar 9741), Japan, Honshu, Aichi Prefecture, Okazaki-shi, Okuyamada-cho, Mt. Murazumi-yama, alt. 200–250 m, 5 May 2011, coll. Kiyoto Ogata. Paratypes, 1♀ (NSMT-Ar 9742), same data as holotype; 2♀♀ and 2♂♂ (NSMT-Ar 9743), same data as holotype.

##### Diagnosis.

The genital characters of *Solenysa ogatai* are very similar to those of *Solenysa partibilis* (Figs 3C–F, 6C–D). The male palp is diagnosed by the posterior branch of the lamella characteristica with two long free ends, the longer one in *Solenysa ogatai* is sigmoid curved in ventral view (Fig. 3C), almost a circle in anterior view (Fig. 3E), while in *Solenysa partibilis* L-curved in ventral view (Fig. 3D), half circle in anterior view (Fig. 3F). The epigyne can be distinguished by the epigynal collar, which is more than four times wider than long in *Solenysa ogatai* (Fig. 5B), but less than twice as wide than long in *Solenysa partibilis* (Tu and Hormiga 2011: fig. 11I).

##### Description.

Somatic characters as in the genus description and for genital characters see Ono (2011).

##### Distribution.

Japan (Honshu, Fig. 7).

#### Solenysa partibilis

Taxon classificationAnimaliaAraneaeLinyphiidae

Tu, Ono & Li, 2007

Figs 1E , 3D, F , 5C–D

Solenysa melloteei : Oi 1960: 153, figs 52–54 (misidentification).
Solenysa partibilis Tu, Ono & Li, 2007: 60, fig. 2A–D; Ono et al. 2009: 332, figs 1092–099; Tu and Hormiga 2011: figs 11I, 12A–H, 13A–H.

##### Type.

Male holotype (NSMT-Ar 2776), Japan, Honshu, Shiga Prefecture, Mt. Ibuki, 35°12'N, 136°12'E, 11 Nov. 1957, coll. Ryoji Oi.

##### Additional Material examined.

3♂♂ and 3♀♀, Japan, Honshu, Tokyo, Omeshi, Mitake, 35°48'N, E139°10.80'E, 17 Oct. 2004, coll. Akihisa Andoh; 3♂♂ and 3♀♀ (CNU-J01), Japan, Houshu, Tokyo, Ome-shi, Mitake, 35°48.08'N, E139°11.15'E, 17 Oct. 2004, coll. Akihisa Andoh; 3♀♀ (CNU-J25), Japan, Honshu, Fukushima Prefecture, Fukushima-shi, Kanayagawa, 37°41.42'N, 140°27.18'E, alt. 190 m, 28 Feb. 2009, coll. Akihisa Andoh; 2♂♂ and 3♀♀ (CNU-J31), Japan, Honshu, Shiga Prefecture, Maibara (base of Mt. Ibuki), Ohshimizu, 35°22.37'N, 136°24.08'E, alt. 190 m, 2 Jun. 2009, coll. Akihisa Andoh; 1♀ (CNU-J33), Japan, Honshu, Niigata Prefecture, Niitsu, Akihayama, 37°47.02'N, 139° 08.32'E, alt. 50 m, 20 Jun. 2009, coll. Akihisa Andoh; 1♀ (CNU-J34), Japan, Honshu, Niigata Prefecture, Niitsu, 37°46'N, 139°08.20'E, alt. 50 m, 20 Jun. 2009, coll. Akihisa Andoh; 2♀♀ (CNU-J35), Japan, Honshu, Akita Prefecture, Akita-shi, Katsurane, 39°39.32'N, 140°05.10'E, alt. 60 m, 2 Jul. 2009, coll. Akihisa Andoh; 4♂♂ and 7♀♀ (CNU-J36), Japan, Honshu, Miyagi Prefecture, Sendai, Mt.Takamori, 38°19.03'N, 140°56.17'E, 23 Aug. 2009, coll. Akihisa Andoh; 2♂♂ and 1♀ (CNU-J39), Japan, Honshu, Tokyo, Ome, Yugi, 35°48.18'N, 139°11.98'E, alt. 240 m, 12 Sept. 2009, coll. Akihisa Andoh.

##### Diagnosis.

See diagnosis for *Solenysa ogatai*.

##### Description.

Somatic characters as in the genus description (Fig. 1E, see also Tu and Li 2006, Tu and Hormiga 2011), and genital characters see the descriptions by Tu et al. (2007) and Tu and Hormiga (2011).

##### Distribution.

Japan (Honshu, Fig. 7).

#### Solenysa reflexilis

Taxon classificationAnimaliaAraneaeLinyphiidae

Tu, Ono & Li, 2007

Fig. 4E–F

Solenysa reflexilis Tu, Ono & Li, 2007: 58, fig. 1A–H; Ono et al. 2009: 332, figs 1100–1104.

##### Types.

Male holotype (NSMT-Ar 3905), Japan, Kyushu, Kumamato Prefecture, Kuma-gun, Itsukimura, Shimo-kajiwara, 32°12'N, 130°30'E, 27 Oct. 1981, coll. Teruo Irie. Paratype, 1♂ and 2♀♀, same data as holotype.

##### Additional material examined.

2♀♀ (CNU-J28), Japan, Kyushu, Kumamoto Prefecture, Itsuki, Touji, 32°23.63'N, 130°49.67'E, alt. 310 m, 27 Apr. 2009, coll. Akihisa Andoh; 1♀ (CNU-J29), Japan, Kyushu, Kumamoto Prefecture, Sagara, 32°8.67'N, 130°51.53'E, alt. 590 m, 28 Apr. 2009, coll. Akihisa Andoh; 1♀ (CNU-J30), Japan, Kyushu, Kumamoto Prefecture, Sagara, Nagae, 32°18.67'N, 130°51.53'E, alt. 170 m, 22 Jul. 2006, coll. Akihisa Andoh.

##### Diagnosis.

See the diagnosis for *Solenysa macrodonta* sp. n.

##### Description.

Somatic characters as in the genus description (see also Tu and Li 2006, Tu and Hormiga 2011), and genital characters as in the description by Tu et al. (2007).

##### Distribution.

Japan (Kyushu, Fig. 7).

#### Solenysa trunciformis sp. n.

Taxon classificationAnimaliaAraneaeLinyphiidae

http://zoobank.org/AD5A06F1-6832-4FFB-B5E0-652F483A491A

Figs 1A–D , 3B , 5E–F

Solenysa melloteei : Tu and Li 2006: 91, figs 21–30; Tu and Hormiga 2011: 499, fig. 8A–I (misidentification).

##### Types.

Male holotype (CNU-J26), Japan, Honshu, Wakayama Prefecture, Shirahama, Tondazaka, 33°37.53'N, 135°25.35'E, alt. 310 m, 31 Mar. 2009, coll. Akihisa Andoh. Paratypes, 2♂♂ and 9♀♀, same data as holotype; 1♂ and 1♀ (CNU-J23), Japan, Shikoku, Kagawa Prefecture, Takamatsu, Nishi-ueda, 34°13.22'N, 134°04.62'E, alt. 130 m, 19 Jul. 2008, coll. Akihisa Andoh; 1♂ and 3♀♀ (CNU-J05), Japan, Honshu, Wakayama Prefecture, Susami-cho, Esuzaki, 33°30'N, 135°34.20'E, 24 Aug. 1981, coll. Yoshito Ishii.

##### Diagnosis.

The male palpal characters of *Solenysa trunciformis* sp. n. (Figs 1C, 3B, 6F) are similar to those of *Solenysa macrodonta* sp. n. and *Solenysa refrexilis*; to distinguish them see the diagnosis for *Solenysa macrodonta* sp. n. The female is distinguished by the apple-shaped epigyne with a rectangular epigynal collar (Fig. 5F).

##### Description.

Somatic characters as in the genus description and genital characters as in the descriptions for *Solenysa mellotteei* by Tu and Li (2006) and Tu and Hormiga (2011).

##### Etymology.

The species name comes from the Latin ‘*trunciformis*’ in reference to truncate apex of anterior protrusion in front of median terminal apophysis (Fig. 1C).

##### Distribution.

Japan (Honshu, Shikoku, Fig. 7).

### Key to *Solenysa* species from the islands of Japan

**Table:** 

1	Male	**2**
–	Female	**7**
2	LC_2_ with a forked apex, LC_3_ unbranched (Fig. 3A)	**3**
–	LC_2_ with a sharp apex, LC_3_ includes two parts (Fig. 3C)	**5**
3	MTA with serrated margin (Fig. 3A)	***Solenysa macrodonta* sp. n.**
–	MTA with smooth margin and two anterior protrusions (Fig. 3B)	**4**
4	First protrusion truncate (Fig. 3B)	***Solenysa trunciformis* sp. n.**
–	First protrusion pointed (Tu et al. 2007, fig. 1D)	***Solenysa reflexilis***
5	Anterior part of LC_3_ flag-shaped (Fig. 2B)	***Solenysa mellotteei***
–	Anterior part of LC_3_ spike-shaped (Fig. 3C–D)	**6**
6	Posterior part of LC_3_ S-curved (Fig. 3C)	***Solenysa ogatai***
–	Posterior part of LC_3_ L-curved (Fig. 3D)	***Solenysa partibilis***
7	Dorsal plate almost as wide as long (Fig. 4B)	**8**
–	Dorsal plate wider than long (Fig. 5B)	**9**
8	Epigynal collar inversed triangular (Fig. 4A)	***Solenysa mellotteei***
–	Epigynal collar rectangular (Fig. 5F)	***Solenysa trunciformis* sp. n.**
9	Posterior margin of epigyne centrally incised (Fig. 4A–C)	**10**
–	Posterior margin of epigyne straight (Fig. 4E)	***Solenysa reflexilis***
10	Maximum width at anterior part (Fig. 5A)	**11**
–	Maximum width in middle (Fig. 4C)	***Solenysa macrodonta* sp. n.**
11	Epigynal collar more than four times wider than long (Fig. 5B)	***Solenysa ogatai***
–	Epigynal collar less twice wider than long (Tu and Hormiga 2011: fig. 11I)	***Solenysa partibilis***

## Supplementary Material

XML Treatment for Solenysa

XML Treatment for Solenysa mellotteei group

XML Treatment for Solenysa mellotteei

XML Treatment for Solenysa macrodonta

XML Treatment for Solenysa ogatai

XML Treatment for Solenysa partibilis

XML Treatment for Solenysa reflexilis

XML Treatment for Solenysa trunciformis
